# Design and Performance Validation of 4D Radar ICP-Integrated Navigation with Stochastic Cloning Augmentation

**DOI:** 10.3390/s26051660

**Published:** 2026-03-05

**Authors:** Hyeongseob Shin, Dongha Kwon, Sangkyung Sung

**Affiliations:** 1 Department of Aerospace Information Engineering, Konkuk University, Seoul 05029, Republic of Korea; shinhyung12@konkuk.ac.kr; 2 Convergence Major of Future Mobility, Department of Aerospace and Mobility Engineering, Konkuk University, Seoul 05029, Republic of Korea; kglenn1601@konkuk.ac.kr; 3 Department of Aerospace and Mobility Engineering, Konkuk University, Seoul 05029, Republic of Korea

**Keywords:** radar, radar-inertial odometry, iterative closest point

## Abstract

**Highlights:**

**What are the main findings?**

- An ICP-integrated radar-inertial odometry framework is proposed for 4D imaging radar, integrating relative pose from radar ICP with Doppler-based ego-velocity in a consistent EKF framework.
- Experiments on various datasets demonstrate that integrating ICP and Doppler velocity achieves higher localization accuracy and robustness than ICP-only or velocity-only approaches.

**What are the implications of the main findings?**

- Integrating point cloud-based spatial constraints with Doppler velocity significantly improves estimation stability under sparse, noisy, and dynamically changing radar environments.
- The modular ICP-integrated observation model enables scalable and flexible adoption of future ICP algorithms, supporting reliable real-time navigation using 4D imaging radar.

**Abstract:**

Automotive radar has emerged as a pivotal technology for navigation in GNSS-denied environments, offering superior robustness to adverse weather and fluctuating lighting conditions compared to vision or LiDAR-based sensors. Despite these advantages, the inherent sparsity and noise of radar measurements often lead to degraded estimation accuracy and system reliability. To address these challenges, various radar-based localization frameworks have been explored, ranging from optimization-based and Extended Kalman Filter (EKF) approaches fused with Inertial Measurement Units (IMUs) to point cloud registration techniques like Iterative Closest Point (ICP). While filter-based methods are favored in multi-sensor fusion for their proven stability, ICP is widely utilized for high-precision pose estimation in point-cloud-centric systems. In this study, we propose a novel Radar-Inertial Odometry (RIO) framework that synergistically integrates ICP-based relative pose estimation with model-based sensor fusion. The proposed methodology leverages relative transformations derived from ICP alongside ego-velocity estimations obtained from radar Doppler measurements. To effectively incorporate relative ICP constraints, a stochastic cloning technique is implemented to augment previous states and their associated covariances, ensuring that the uncertainty of historical poses is explicitly accounted for. The performance of the proposed method is validated using public open-source datasets, demonstrating higher localization accuracy and more consistent performance compared to existing algorithms used for comparison.

## 1. Introduction

With the recent increase in high-rise buildings and complex urban infrastructures in city centers, regions where the Global Navigation Satellite System (GNSS) cannot be reliably used have rapidly expanded. Consequently, autonomous navigation technologies utilizing sensors such as cameras and LiDAR have been actively studied. Camera sensors offer cost-effectiveness and low power consumption, while LiDAR provides direct distance measurements with centimeter-level precision. Owing to these advantages, both sensors have become core components of autonomous driving systems. However, their performance degrades significantly under adverse weather conditions (e.g., rain, snow, fog) or in low-light environments [1,2,3,4]. To achieve fully autonomous navigation, a sensor with higher environmental robustness is therefore required.

Against this backdrop, radar sensors have recently gained attention as a promising next-generation alternative. Unlike LiDAR, radar sensors use radio waves, making them less susceptible to environmental factors such as lighting and weather. At the same time, they share the benefits of cameras in terms of low power consumption and cost efficiency. Moreover, radar can directly measure the Doppler velocity, enabling vehicle speed estimation using a single sensor. Nevertheless, radar data are inherently sparse and noisy, resulting in lower precision compared to LiDAR or camera data under similar conditions. For this reason, research on achieving high-precision localization using radar sensors has been actively pursued [5,6,7,8,9].

Typical approaches for radar-only navigation include point cloud registration techniques such as Iterative Closest Point (ICP) and Normal Distribution Transform (NDT) [10,11,12,13,14,15]. As these methods have evolved, recent studies [12] have reported that their accuracy approaches that of LiDAR-based registration algorithms. However, radar sensors alone still face challenges in obtaining dense, high-resolution data, and due to their inherent characteristics, navigation errors can diverge in the event of pose estimation failure. To overcome these issues, extensive research has focused on sensor fusion methods combining multiple modalities.

In particular, the Inertial Measurement Unit (IMU) is robust against external disturbances and provides high-frequency measurements, making it an ideal complement to radar for building resilient and accurate navigation systems. To this end, recent studies [16,17,18,19,20,21] have proposed various optimization- and Extended Kalman Filter (EKF)-based radar-IMU fusion approaches. While optimization-based methods can generally achieve higher accuracy, they suffer from heavy computational loads, making real-time implementation challenging. Conversely, EKF-based methods are computationally efficient, suitable for real-time applications, and have been widely studied. Previous works [22] have reported that an invariant EKF can achieve accuracy comparable to optimization-based methods in both 2D and 3D environments while maintaining computational efficiency.

Despite these advantages, there have been relatively few studies directly applying EKF to Radar-Inertial Odometry (RIO). Representative EKF-based RIO studies include [23,24,25,26,27]. Both [23] and [24] employ lightweight 3D FMCW radar. The study [23] adopts a loosely coupled architecture that integrates only INS with radar ego-velocity, yet achieves strong performance. In contrast, ref. [24] tightly couples the IMU and radar, proposing a point-matching method that remains robust under noisy and sparse radar point clouds and leverages both range and velocity models to deliver accurate estimation. However, these methods were designed for 3D FMCW radar and exhibit limitations when transferred to 4D imaging radar. Specifically, the velocity-centric design of [23] does not fully exploit the richer point clouds available from 4D imaging radar, while the point-matching pipeline in [24] must operate on point sets that are at least about five times larger than those from 3D FMCW radar, substantially increasing computational complexity. Consequently, in 4D imaging radar settings, redesigns or lightweight data-association strategies that account for the sensor’s data density and characteristics may yield meaningful performance gains.

Therefore, this study proposes a novel algorithm that integrates high-precision navigation information from radar-based ICP with IMU data through an EKF framework. The proposed algorithm offers high scalability, allowing flexible integration with various ICP algorithms. Generally, ICP provides the relative pose between radar frames, and if this relative pose is accumulated over time (i.e., composed sequentially), integrating it with inertial navigation becomes relatively straightforward. However, handling relative measurements in this way makes it difficult to account for the uncertainty of previous steps during measurement accumulation.

To address this limitation, this study adapts the stochastic cloning technique, previously proposed in the related studies [24,28], enabling direct integration of relative measurements from ICP within the integrated navigation framework. Furthermore, by integrating Doppler velocity measurements through the ego-velocity model, the proposed navigation framework can take advantage of position, velocity, and attitude measurements in a simultaneous way.

Remainder of this paper is organized as follows. Section 2 introduces a description of the entire system. Section 3 presents the proposed navigation framework, including the measurement model. Section 4 describes the open datasets used and reports the evaluation results. Finally, Section 5 concludes the paper and discusses directions for future work.

## 2. System Description

The state vector employed in this work explicitly includes the pose at the previous time step to consistently accommodate the relative measurements provided by radar ICP. This design enables direct modeling of ICP-based relative observations in the measurement model. Using relative measurements requires both the current state and the state at the previous epoch. However, merely buffering the previous pose is insufficient because it is difficult to properly account for the uncertainty associated with past measurements. To address this issue, we adopt stochastic cloning, originally proposed in [28] and applied to radar relative measurements in [24]. Concretely, upon the arrival of a radar scan, the pose at that instant together with its covariance is augmented as a clone in the EKF state vector so that, when the ICP-derived relative transform (translation and rotation) is used as a measurement, the uncertainties of the cloned state and the current state are naturally considered in the update. Because the ICP module relies only on two consecutive scans, the augmented state is limited to the immediately preceding pose and covariance. After the measurement update, the clone is refreshed to the latest reference.

Although ICP with radar measurements has historically suffered from degraded registration performance in sparse 3D FMCW radar point clouds, recent advances in preprocessing, feature extraction and robust data association—along with the substantially higher point density of 4D imaging radar—have yielded registration accuracies that approach those of LiDAR-based ICP, and in certain conditions comparable performance has been reported [12]. Motivated by these trends, we adopted radar ICP as the measurement module, balancing accuracy with real-time feasibility [12]. The proposed framework is not tied to any specific ICP variant and can seamlessly integrate a range of state-of-the-art formulations in a unified manner.

**Figure 1:**
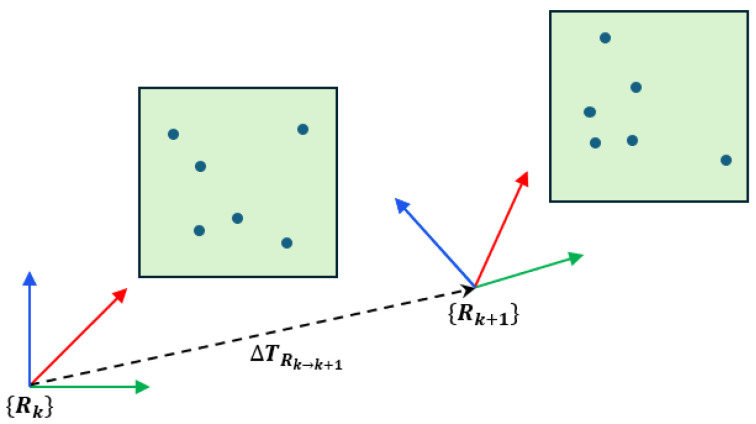
ICP registration between two successive radar scans.

While conventions may vary across implementations, in this paper, we estimate the relative pose between the radar body frames $r_{k}$ and $r_{k+1}$ by aligning two consecutive radar scans with ICP, yielding $\Delta T_{r_{k}\to r_{k+1}}$. The point-to-point ICP minimizes (2) using scan correspondences, and we emphasize that the result is the relative transformation between sensor body frames, not a local pose. This procedure is illustrated schematically in Figure 1, which intuitively depicts the ICP configuration adopted in this study.

Finally, the optimization problem used in this study for radar ICP is formulated as in (1), which extends the conventional point-to-point ICP by incorporating a Doppler velocity constraint.

(1) $$T^{*}=\underset{T}{\mathrm{argmin}}\left(1-\gamma \right)E_{t}\left(T\right)+\gamma E_{v}\left(T\right)$$

The term with subscript t denotes the point-to-point constraint, while the term with subscript v denotes the Doppler velocity constraint. The relative contribution of the two terms is controlled by the scalar weight γ. When γ=0, the objective reduces to a purely point-to-point form. The function ρ(·) is the Geman–McClure kernel, used as a robust loss to mitigate the influence of outliers and dynamic objects in radar returns.

(2) $$E_{t}\left(T\right)=\sum_{k=1}^{N}\rho_{t}\left(\left\|q_{k}-Tp_{k}\right\|\right)$$

(3) $$E_{v}\left(T\right)=\sum_{k=1}^{N}\rho_{t}\left(\left\|v_{D_{k}}-v_{{est}_{k}}\left(T\right)\right\|\right)$$

As in (3), the Doppler velocity constraint is formulated to minimize the discrepancy between the estimated and measured Doppler velocity.

(4) vestkT=−dk·(vtT+vθT×tsV)v

The estimated Doppler velocity is defined as in (4), obtained by projecting the rigid-body velocity vector—composed of the radar’s linear and angular velocities computed from Euler’s rigid-body motion equations—onto the radial direction.

The proposed framework is based on a scan-to-scan ICP scheme. Due to this structural characteristic, excessive point removal may reduce the number of effective correspondences available for registration, thereby degrading convergence performance and potentially causing divergence of the ICP algorithm. Accordingly, no explicit feature extraction was performed during the preprocessing stage, and the geometric structure of the point cloud was preserved as much as possible. Outlier rejection was conducted exclusively using a RANSAC-based robust estimation method. The main parameters used in this study are summarized in Table 1.

## 3. Navigation Framework

The navigation framework in this work employs an IMU and a radar. To ensure the convergence of the ICP registration, we use a 4D imaging radar sensor that provides at least 200 points per scan. An EKF serves as the navigation filter, with IMU measurements used for state propagation and radar measurements used for the measurement update.

**Figure 2:**
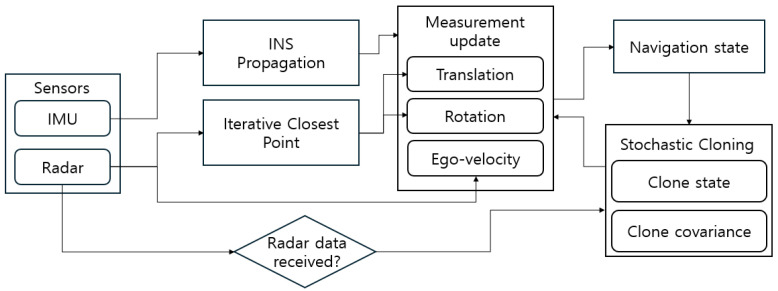
Proposed navigation framework chart.

Figure 2 summarizes the overall pipeline of the proposed framework, and Algorithm 1 presents the corresponding step-by-step execution procedure.
**Algorithm 1** Runtime procedure of the proposed framework.1:**Initialization:** ${\hat{x}}_{0|0},{\hat{x}}_{0|0},\;Q,\;R,\;ICP\;parameters$2:**for** *k = 1, 2, 3, …***do**3:

       ${\hat{x}}_{k|k-1}=f({\hat{x}}_{k-1|k-1})$
4:

       $\Sigma_{k|k-1}=F_{k}\Sigma_{k-1|k-1}{F_{k}}^{T}+G_{k}Q{G_{k}}^{T}$
5:
       **if** a new radar scan is available **then**6:

                **if** first radar scan **then**7:



   ${\hat{p}}^{p}\leftarrow {\hat{p}}_{k|k-1}^{c}$
8:



   ${\hat{q}}^{p}\leftarrow {\hat{q}}_{k|k-1}^{c}$
9:


   $\hat{x}$ $\leftarrow \;{\left[{\hat{x}}_{k|k-1},{\hat{p}}^{p},{\hat{q}}^{p}\right]}^{T}$10:


   $\Sigma_{3,k|k-1}$ $\leftarrow \;\Sigma_{1,k|k-1}$11:



   $\Sigma_{k|k-1}\leftarrow \left[\begin{array}{ccc} \Sigma_{1,k|k-1} & \Sigma_{1,2,k|k-1} & \Sigma_{1,k|k-1}\\ \Sigma_{2,1,k|k-1} & \Sigma_{2,k|k-1} & \Sigma_{2,1,k|k-1}\\ \Sigma_{1,k|k-1} & \Sigma_{1,2,k|k-1} & \Sigma_{3,k|k-1} \end{array}\right]$
12:


   Set current scan as ICP source13:


                **else**

14:


   Set current scan as ICP target15:


   Compute ($R_{k},\;t_{k}$) using ICP16:


   Compute $v_{k}^{r}$ using LSQ17:



   ${\tilde{r}}_{p,k}\leftarrow t_{k}-{\left({\hat{R}}_{k|k-1}^{c}\right)}^{T}({\hat{p}}^{p}-{\hat{p}}_{k|k-1}^{c})$
18:



   ${\tilde{r}}_{\theta ,k}\leftarrow LogSO3(R_{k}{\left({\hat{R}}_{k|k-1}^{p}\right)}^{T}{\hat{R}}_{k|k-1}^{c})$
19:



   ${\tilde{r}}_{v,k}\leftarrow v_{k}^{r}-({\left(R_{r}^{b}\right)}^{T}\left({\left[\omega_{k,b}-{\hat{b}}_{k,gyro}^{b}\right]}_{\times }l_{r}^{b}\right)+{\left({\hat{R}}_{k|k-1}^{c}\right)}^{T}{\hat{v}}_{k|k-1}^{c})$
20:



   ${\tilde{z}}_{k}=\left[\begin{array}{c} {\tilde{r}}_{p,k}\\ {\tilde{r}}_{\theta ,k}\\ {\tilde{r}}_{v,k} \end{array}\right]$
21:



   $K=\Sigma_{k|k-1}H_{k}^{T}{\left(H_{k}\Sigma_{k|k-1}H_{k}^{T}+R\right)}^{-1}$
22:



   ${\hat{x}}_{k|k}\leftarrow {\hat{x}}_{k|k-1}+K{\tilde{z}}_{k}$
23:



   $\Sigma_{k|k}\leftarrow \left(I-KH_{k}\right)\Sigma_{k|k-1}\;$
24:


   Set target scan as next ICP source25:



   ${\hat{p}}^{p}\leftarrow {\hat{p}}_{k|k}^{c}$
26:



   ${\hat{q}}^{p}\leftarrow {\hat{q}}_{k|k}^{c}$
27:


                **end if**
28:

       **end if**
29:**end for**

### 3.1. Propagation

The state vector in this work is defined as follows: it comprises the current position, attitude (quaternion), velocity, accelerometer bias, and gyroscope bias, together with the past position and attitude. Position, attitude, and velocity are expressed in the navigation frame, whereas the accelerometer and gyroscope bias are expressed in the body frame. The superscript c denotes the current time, and the subscript p denotes the past time.

(5) $$x={\left[x^{c},x^{p}\right]}^{T}={\left[\left[\begin{array}{ccccc} p_{n}^{c} & q_{n}^{c} & v_{n}^{c} & b_{acc}^{c} & b_{gyro}^{c} \end{array}\right],\left[\begin{array}{cc} p_{n}^{p} & q_{n}^{p} \end{array}\right]\right]}^{T}$$

In this paper, we adopt an error-state formulation and define the following error-state vector based on (5).

(6) $$\tilde{x}={\left[{\tilde{x}}^{c},{\tilde{x}}^{p}\right]}^{T}={\left[\left[\begin{array}{ccccc} {\tilde{p}}_{n}^{c} & {\tilde{\theta }}_{n}^{c} & {\tilde{v}}_{n}^{c} & {\tilde{b}}_{acc}^{c} & {\tilde{b}}_{gyro}^{c} \end{array}\right],\left[\begin{array}{cc} {\tilde{p}}_{n}^{p} & {\tilde{\theta }}_{n}^{p} \end{array}\right]\right]}^{T}$$

As noted in Section 2 (System Description), the “past state” ${\left(\cdot \right)}^{p}$ in this paper refers to the state augmented via stochastic cloning at the time the previous measurement arrived. When a new measurement arrives, the cloned state is updated. This design enables the navigation filter to consistently exploit the relative measurements from ICP by jointly accounting for the uncertainties of both the past and current states. 

The basic formulation follows [24], with some revised notation. Since the past state is not propagated, only the current states are propagated according to (7).

(7) $$x_{k+1}^{-}=f\left(x_{k},u\right)=\left[\begin{array}{c} p_{k}^{n}+v_{k}^{n}\cdot dt+\frac{1}{2}\left(C_{b}^{n}\cdot A_{b}-g_{n}\right)\cdot dt\\ q_{k}+{\dot{q}}_{k}\cdot dt\\ v_{k}^{n}+\left(C_{b}^{n}\cdot A_{b}-g_{n}\right)\cdot dt\\ b_{acc,k}\\ b_{gyro,k} \end{array}\right]$$

Since the cloned state does not propagate over time, the state transition matrix Φ for that block is the identity matrix, and the noise input matrix G is a zero matrix. The system-wide matrices, Φ and G, can be written in block matrix form as shown.

(8) $$\Phi_{augmented}=\left[\begin{array}{cc} \Phi_{c} & 0\\ 0 & I \end{array}\right],G_{augmented}=\left[\begin{array}{c} G_{c}\\ 0 \end{array}\right]$$

Along with the state, the same procedure is applied to the covariance: Upon receipt of a new radar measurement, the corresponding covariance matrix is replicated and augmented

(9) $$\Sigma_{1}=\left[\begin{array}{cc} \Sigma_{p^{c}} & \Sigma_{p^{c},q^{c}}\\ \Sigma_{q^{c},p^{c}} & \Sigma_{q^{c}} \end{array}\right]$$

Equation (9) denotes the covariance of the pose at the current time, while (10) denotes the covariance of the remaining state at the current time.

(10) $$\Sigma_{2}=\left[\begin{array}{ccc} \Sigma_{v} & \Sigma_{{v,b}_{a}} & \Sigma_{v,b_{g}}\\ \Sigma_{b_{a},v} & \Sigma_{b_{a}} & \Sigma_{b_{a},b_{g}}\\ \Sigma_{b_{g},v} & \Sigma_{b_{g},b_{a}} & \Sigma_{b_{g}} \end{array}\right]$$

(11) $$\Sigma_{3}=\left[\begin{array}{cc} \Sigma_{p^{p}} & \Sigma_{p^{p},q^{p}}\\ \Sigma_{q^{p},p^{p}} & \Sigma_{q^{p}} \end{array}\right]$$

The covariance of the past (i.e., cloned) state can be written as (11), and at the cloning instant (i.e., when the radar measurement is received) it is identical to that in (9).

(12) $$\Sigma =\left[\begin{array}{ccc} \Sigma_{1} & \Sigma_{1,2} & \Sigma_{1}\\ \Sigma_{2,1} & \Sigma_{2} & \Sigma_{2,1}\\ \Sigma_{1} & \Sigma_{1,2} & \Sigma_{3} \end{array}\right]$$

Finally, the augmented covariance at the cloning instant can be written as in (12).

### 3.2. ICP Measurement Model

One of the measurement models proposed in this study is designed to correct the propagation error of inertial navigation by utilizing the relative pose estimated from ICP alignment between consecutive radar point clouds. By performing ICP between two successive radar scans, the relative position and orientation between the previous and current frames are estimated and integrated into the correction step as measurements. Since such relative measurements depend on states of two different time steps, the state vector is augmented to maintain the cross-correlation between the past and current states within the filter. This formulation ensures that the ICP-based relative transformation is consistently reflected in the filter and effectively compensates for the accumulated drift in inertial propagation.

The relative transformation obtained from ICP is defined in the sensor coordinate frame and then converted to the navigation frame to construct the measurement residual. The residual is defined as the difference between the predicted relative transformation and the ICP-derived transformation, and the standard EKF update equations are applied. During the update process, only the current state is propagated by the inertial sensor model, while the past state remains static, allowing the information from the relative transformation to be distributed consistently across the two states. This mitigates the effect of accumulated attitude errors on position estimation.

For a real-time operation, ICP is not performed on every radar frame. Instead, a gating process based on quality indicators such as the number of correspondences, RMS residual, and convergence iterations is applied to reject unreliable measurements. State augmentation and covariance updates are performed only for the accepted measurements, followed by simultaneous correction of position and orientation during the state estimation process.

The transformation obtained from the ICP results in (13) can be decomposed into translational and rotational components, which are presented in (14) and (15), respectively.

(13) $$T_{ICP}=\left[\begin{array}{cc} R_{ICP} & t_{ICP}\\ 0 & 1 \end{array}\right]\in SE\left(3\right)$$

(14) $$R_{ICP}=\left[\begin{array}{ccc} r_{11} & r_{12} & r_{13}\\ r_{21} & r_{22} & r_{23}\\ r_{31} & r_{32} & r_{33} \end{array}\right]\in SO\left(3\right)$$

(15) $$t_{ICP}=\left[\begin{array}{c} t_{x}\\ t_{y}\\ t_{z} \end{array}\right]\in R^{3}$$

#### 3.2.1. Translation Model

The geometric model for the translation obtained from ICP is given by (16), and it can be expressed in terms of the error state as (17).

(16) tICP=RcbnTpnp−pnc

(17) tICP=R^ cbnexpθ~nc×Tp^np+p~np−p^nc+p~nc=exp−θ~nc×R^ cbnTp^np−p^nc+p~np−p~nc

The difference between the past and current positions is expressed as in (18) and using the first-order small angle approximation [29], it can be written as (19).

(18) $$∆{\hat{p}}_{n}≔{\hat{p}}_{n}^{p}-{\hat{p}}_{n}^{c}$$

(19) $$exp\left({-\left[{\tilde{\theta }}_{n}^{c}\right]}_{\times }\right)\approx I_{3}-{\left[{\tilde{\theta }}_{n}^{c}\right]}_{\times }$$

By substituting (18) and (19) into (17), we obtain (20).

(20) tICP≈I3−θ~nc×R^ cbnT∆p^n+p~np−p~nc

After expanding (20) and neglecting the second-order terms, we obtain (21).

(21) tICP≈R^cbnT∆pn+p~np−p~nc−θ~nc×R^cbnT∆p^n

At this stage, by rewriting the term involving ${\tilde{\theta }}_{n}^{c}$ in the relation as a vector expression [29], (22) is obtained.

(22) −θ~nc×R^ cbnT∆p^n=R^ cbnT∆p^n×θ~nc

By substituting (22) into (21), we finally obtain

(23) tICP≈R^ cbnT∆pn+p~np−p~nc+R^ cbnT∆p^n×θ~nc

Finally, the observation matrix of the EKF for the translation model is given by (24).

(24) HT=−R^cbnTR^cbnT∆p^n×03×9R^cbnT03×33×21

#### 3.2.2. Rotation Model

The geometric model of the rotation measurement obtained from ICP is given in (25). Since this measurement model lies in SO (3), it is first mapped to the tangent space by the log mapping and then converted into a vector in $R^{3}$ via the vee operator. Using the error-state formulation, this can be expressed as in (26).

(25) R*radar=RcbnTRpbn∈SO3

(26) Rradar=logRcbnTRpbn∨=logR^cbnexpθ~nc×TR^pbnexpθ~np×∨

By expanding the transpose in (26), the expression can be rewritten as (27).

(27) Rradar=logexp−θ~nc×R^ cbnTR^ pbnexpθ~np×∨

Under the assumption of small rotations of the sensor platform, the expression can be approximated as R^cbnTR^pbn≈I, and therefore, (27) can be rewritten as (28).

(28) $$R_{radar}\approx {\left(log\left(exp{\left(\left[-{\tilde{\theta }}_{n}^{c}\right]\right)}_{\times }exp{\left(\left[{\tilde{\theta }}_{n}^{p}\right]\right)}_{\times }\right)\right)}^{\vee }$$

Equation (28) is transformed into (29) by applying the first-order Baker–Campbell–Hausdorff (BCH) approximation [29].

(29) $$R_{radar}\approx {\left(log\left(exp{\left(\left[-{\tilde{\theta }}_{n}^{c}+{\tilde{\theta }}_{n}^{p}\right]\right)}_{\times }\right)\right)}^{\vee }$$

By canceling the log and exp mappings in (29), we obtain (30), and by removing the skew-symmetric operator and the Vee operator, the measurement model reduces to (31).

(30) $$R_{radar}\approx {\left({\left(\left[-{\tilde{\theta }}_{n}^{c}+{\tilde{\theta }}_{n}^{p}\right]\right)}_{\times }\right)}^{\vee }$$

(31) $$R_{radar}\approx -{\tilde{\theta }}_{n}^{c}+{\tilde{\theta }}_{n}^{p}$$

Finally, the observation model of the EKF for the rotation obtained from ICP can be expressed as (32).

(32) $$H_{R}={\left[\begin{array}{ccc} 0_{3\times 3} & {-I}_{3\times 3} & \begin{array}{cc} 0_{3\times 12} & I_{3\times 3} \end{array} \end{array}\right]}_{3\times 21}$$

### 3.3. Ego-Velocity Measurement Model

The radar-based ego-velocity measurement model used in this study follows the RIO framework, previously employed in [23]. Let $v_{n}$ denote the platform velocity in the navigation frame, $R_{b}^{n}$ the rotation matrix from the navigation frame to the body frame, and $\omega_{b}$ the IMU angular rate measurement expressed in the body frame. The geometric relationship between the radar and the IMU in the body frame is characterized by the lever arm $l_{r}^{b}$ and the fixed rotation $C_{r}^{b}$ from the body frame to the radar frame. Denoting the gyroscope bias $b_{gyro}^{b}$, the predicted ego-velocity in the radar frame is then given by the following nonlinear measurement model in (33).

(33) $$v^{r}={\left(R_{r}^{b}\right)}^{T}\left(\left[\omega_{b}-b_{gyro}^{b}\right]\right)\times l_{r}^{b}+{\left(R_{b}^{n}\right)}^{T}v^{n}$$

**Figure 3:**
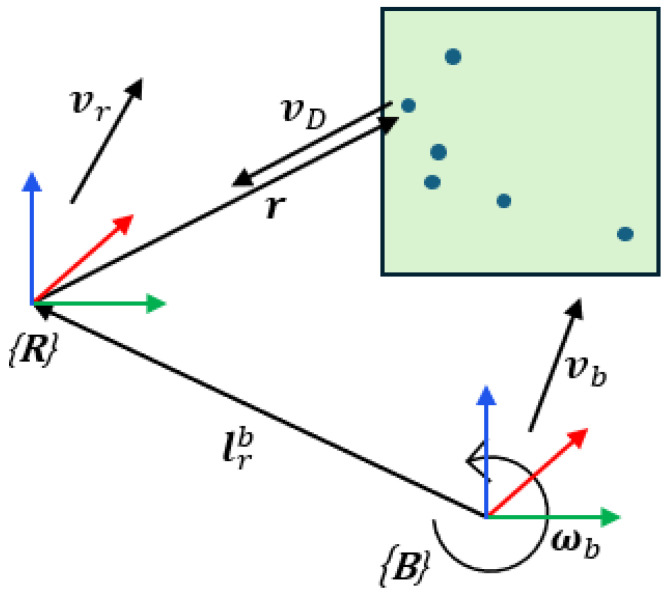
Geometry of radar ego-velocity estimation.

Equation (33) is derived from the Euler rigid-body motion equation, and the ego-velocity measurement can be obtained by applying a least-squares method to the measured Doppler velocities and the corresponding point cloud coordinates. This relationship is illustrated schematically in Figure 3.

The observation matrix for the radar ego-velocity measurement with respect to the full error state $\tilde{x}$ can be written as (35), which is obtained from (34).

(34) R=RrbTR^ cbnT

(35) $$H_{V}={\left[\begin{array}{ccc} 0_{3\times 3} & R{\left[{\hat{v}}_{n}^{c}\right]}_{\times } & \begin{array}{cccc} R & 0_{3\times 3} & {\left(R_{r}^{b}\right)}^{T}{\left[l_{r}^{b}\right]}_{\times } & 0_{3\times 6} \end{array} \end{array}\right]}_{3\times 21}$$

And, using this observation matrix, the standard EKF measurement update is performed so that the radar-based ego-velocity information is integrated into the inertial navigation frame.

## 4. Evaluation

### 4.1. Experimental Setup

All performance evaluations in this paper were conducted using publicly available datasets. Two datasets were employed: the SNAIL Radar dataset [30] and the HeRCULES dataset [31]. Both datasets commonly use the Continental ARS548 4D imaging radar, which offers the advantage that, despite being collected on different platforms and in different environments, the data are highly comparable in terms of sensor characteristics.

First, the specifications of the Continental ARS548 4D radar (Continental AG, Hanover, Germany), which is included in both datasets, are summarized as follows. The ARS548 is a long-range 4D radar capable of measuring up to 300 m, providing range, azimuth, elevation, and Doppler velocity simultaneously. The measurable Doppler velocity range is configured to be approximately [−400, 200] m/s, enabling stable observation of the relative velocity of high-speed vehicles and surrounding obstacles. The vertical field of view (VFOV) is about 40°, allowing wide coverage of the frontal area of a vehicle. The number of points obtained per scan is roughly 300–400, depending on the environment and radar configuration, and each point is accompanied by range, angle, radar cross section (RCS), and Doppler velocity information. According to the SNAIL and HeRCULES papers [29,30], ARS548 offers a range accuracy of approximately 0.3 m, an azimuth resolution of about 0.2°, and an elevation resolution of about 0.1°. These specifications indicate that, although it is an automotive radar, it provides sufficient precision to be used as a sensor for SLAM and accurate pose estimation. In addition, although the two datasets differ in platform and environment, both adopt a common “4D radar + inertial sensor (INS/IMU)” configuration, making them suitable for validating the radar-IMU fusion algorithm proposed in this study.

In the SNAIL dataset, the high-precision GNSS/INS system Bynav X36D (Bynav Technology, Changsha, China) achieves an RMS horizontal position error of about 0.235 m and an RMS vertical position error of about 0.14 m under a 10 s GNSS Outage, and it includes a dedicated high-performance IMU. As a result, it can maintain stable attitude and velocity estimation even in high-speed driving or environments with GNSS shadowing. Owing to these characteristics, the SNAIL dataset constructs a high-reliability reference trajectory by integrating not only the X36D-based RTK GNSS/INS solution but also a terrestrial laser scanner (TLS)-based LiDAR-Inertial alignment result. This reference trajectory is used in this paper to evaluate the position and attitude errors of the proposed algorithm.

In contrast, the HeRCULES dataset uses an Xsens MTi-300 IMU (Xsens Technologies B.V., Enschede, The Netherlands) mounted on the roof of the vehicle. The MTi-300 is a compact, high-performance 9-axis IMU with an attitude estimation accuracy of approximately 0.2° RMS in roll/pitch and about 1° RMS in yaw/heading. Furthermore, the HeRCULES dataset uses, in addition to the MTi-300, an RTK-GPS receiver (NovAtel Inc., Calgary, AB, Canada) to provide reference trajectories for long-distance driving even in complex urban environments.

**Figure 4:**
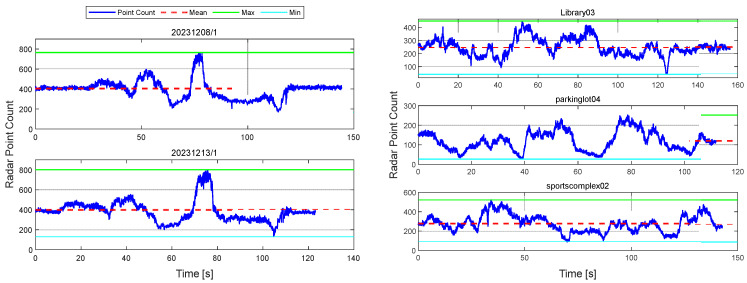
Radar Point Count Over Time Across Multiple Sequences.

Table 2 summarizes the environmental conditions and driving information of the selected sequences. Since each sequence differs in weather, lighting, driving distance, and duration, the corresponding radar point count variations are illustrated in Figure 4.

### 4.2. Evaluation Methodology

To quantitatively evaluate the performance of the proposed method, we employ the open-source trajectory evaluation tool Evo library [31]. Using Evo, we compute several complementary error metrics, including the absolute trajectory error (ATE), which measures the global consistency between the estimated and reference trajectories; the relative position error (RPE), which measures the relative translational error over a fixed interval Δ; and the relative rotation error (RRE), which measures the relative rotational error over the same interval. For RPE and RRE, the reported σ values denote the standard deviation of all per-segment error samples computed along the trajectory. Since the primary objective of this study is accurate localization on the road plane, both the estimated trajectories and the reference trajectories are projected onto a common two-dimensional ground plane prior to evaluation, and all metrics are computed on these 2D projected trajectories.

Furthermore, the above evaluation procedure is applied to the SNAIL and HeRCULES datasets as follows. First, using the SNAIL dataset, we compare the performance of the algorithm that uses only ICP as the measurement model and the proposed algorithm that uses both ICP and ego-velocity as measurements to analyze the benefit of additionally exploiting Doppler velocity information. Subsequently, using the HeRCULES dataset, we compare the proposed algorithm with 4DRadarSLAM [5], KISS-ICP [14] and EKF-LC [23] to assess the impact of integrating ICP-based registration on overall localization performance. The EKF-LC algorithm was implemented by the authors based on an open-source framework.

The main parameter settings of the compared algorithms are summarized as follows: KISS-ICP used the following parameters: $\tau^{0}=2.0$, $\delta_{min}=0.1$, $N_{max}=20$, v=1.0, α=0.5, β=1.5, and $\gamma =1\times 10^{-4}$. 4D Radar SLAM employed ICP-based scan registration with a resolution of 1.0, a maximum correspondence distance of 2.0 m, 64 iterations, and a transformation epsilon of 0.1. The same registration parameters were applied to both the front-end and the back-end. Keyframes were created every 0.5 m/15° in the front-end, while back-end keyframes were spaced at 1.0 m/15°. Loop closure was not used.

### 4.3. Ablation Study

The sequences 20231208/1 and 20231213/1 were selected for experimentation. Both sequences were recorded along the same route using an SUV platform, with the 20231213/1 sequence specifically collected under light rain conditions.

**Figure 5:**
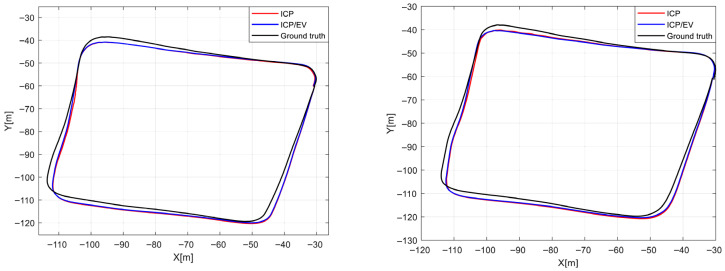
Trajectory comparison by measurement model for SNAIL sequences 20231208/1 (**left**) and 20231213/1 (**right**).

Figure 5 visually presents the navigation results for both sequences, while Table 3 summarizes the corresponding quantitative evaluation metrics. Each entry in the table represents the measurement model used, and EV denotes the ego-velocity model. Underlined values indicate the best-performing method for each metric.

For the 20231208/1 sequence, the algorithm that utilizes only ICP as the measurement model demonstrated superior performance in terms of Absolute Trajectory Error (ATE). In contrast, both algorithms showed comparable performance across other evaluation metrics, each exhibiting its own structural advantages. However, for the 20231213/1 sequence, the algorithm that employed both ICP and ego-velocity as measurement models achieved relatively better performance across most metrics.

This performance discrepancy can be attributed to environmental factors. The 20231213/1 sequence was collected under light rainfall, during which radar reflection intensity was attenuated, and the number of valid points was reduced. Consequently, the reliability of ICP-based registration significantly deteriorated, leading to decreased localization accuracy. Because ICP performance is highly sensitive to the number and spatial distribution of points in the cloud, a reduction in point count directly results in degraded registration quality.

In contrast, ego-velocity estimation based on Doppler measurements is far less affected by the number of detected points, as it relies on the relative radial velocities of targets rather than spatial correspondences. Therefore, even under reduced point density, Doppler-based velocity estimation remains relatively stable, highlighting the robustness of the algorithm that integrates both ICP and ego-velocity models.

This tendency is also evident in the actual data. In the 20231208/1 sequence, an average of 405.4 points per scan (with a minimum of 171 points) were detected, whereas in the 20231213/1 sequence, an average of 399.3 points per scan (with a minimum of 132 points) were observed. These results quantitatively support the claim that rainfall conditions negatively impact the reliability of ICP-based registration.

### 4.4. Validation of the Proposed Framework

In the HeRCULES dataset, three sequences—Library03, Parking Lot04, and Sports Complex02—were selected as experimental subjects. These sequences are larger in scale and exhibit greater environmental diversity than those in the previously used SNAIL dataset. Specifically, they include longer travel distances as well as diverse terrain and reflection characteristics, providing suitable conditions for evaluating the generalization performance of the algorithms. The primary purpose of selecting these datasets is to clearly identify the quantitative performance differences between radar-based methods and the proposed algorithm. In other words, the main focus of this experiment is to analyze how leveraging point clouds based on the ICP (Iterative Closest Point) model affects practical localization accuracy, and to quantitatively evaluate its effectiveness by comparing it with approaches that do not rely on point-cloud registration.

**Figure 6:**
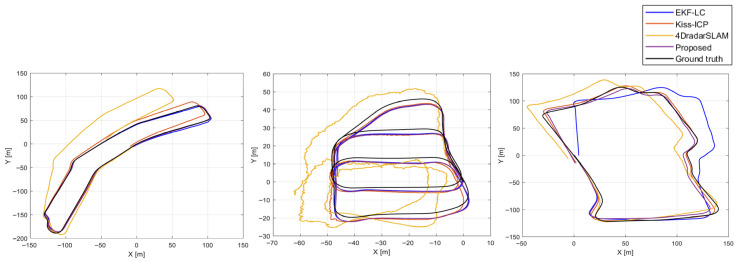
Trajectory comparison by algorithm for HeRCULES sequences Library03 (**left**), Parking Lot04 (**center**), and Sports Complex02 (**right**).

Figure 6 visualizes the driving trajectories of each sequence, enabling an intuitive comparison of the estimated paths across algorithms, while Table 4 summarizes the corresponding quantitative evaluation metrics. Underlined values indicate the best-performing method for each metric. Overall, the results demonstrate that the proposed algorithm consistently outperforms EKF-LC [23] in terms of accuracy. In particular, in the Sports Complex02 sequence, EKF-LC [23] exhibited a clear estimation failure, indicating that the conventional EKF-LC framework [23]—primarily dependent on velocity or inertial information—fails to ensure sufficient stability under complex environmental changes or high-noise conditions. In contrast, the proposed algorithm directly exploits spatial information from point clouds, maintaining both accuracy and robustness even in such challenging environments, thereby demonstrating strong reliability and durability in real-world navigation scenarios.

Moreover, the proposed algorithm also shows superior performance compared to other ICP-based radar navigation approaches. This advantage arises not only from using point clouds, but also from the radar ICP [12] formulation that incorporates Doppler information—an inherent characteristic of radar measurements—as an additional constraint in the ICP registration process. The Doppler constraint effectively restricts inter-frame relative motion, reducing registration ambiguity and mitigating instability caused by sparsity and noise in radar point clouds. Consequently, the proposed method achieves more stable and consistent localization than conventional ICP-based approaches. These results also clearly highlight performance characteristics that depend on sensor type. EKF-LC [23] was originally designed for 3D FMCW radar, for which the number of detectable points is highly limited, making it difficult to sufficiently exploit spatial structural information. Due to this limitation, applying ICP-based point-cloud registration effectively in a 3D FMCW radar setting is challenging, and EKF-LC [23] consequently operates in a manner that largely depends on velocity or inertial information. In such a configuration, yaw is indirectly corrected through velocity constraints, and the observability of yaw-rate bias can become relatively weak. In driving scenarios with limited directional changes, long-term heading drift may accumulate over time. In contrast, 4D imaging radar provides much higher resolution and sufficient point density, allowing the geometric structure of the environment to be represented stably in the form of point clouds. Although this sensor characteristic is highly favorable for ICP-based registration and enables the direct estimation of relative pose constraints, particularly relative yaw, directly applying an algorithm such as EKF-LC [23]—whose core does not explicitly leverage point-cloud registration—to 4D radar prevents the method from fully exploiting the rich spatial information provided by the sensor. As a result, even with 4D radar, such approaches tend to exhibit relatively lower accuracy and stability compared to point cloud-based localization methods. Comparatively, the proposed framework directly utilizes the high-density point clouds from 4D imaging radar and performs radar ICP [12]-based registration by additionally incorporating Doppler constraints, thereby maximizing the sensor’s advantages. This design effectively improves registration quality and directly reflects point-cloud-based spatial information in pose estimation, enabling simultaneous achievement of high-precision and high-reliability localization.

### 4.5. Computational Performance Analysis

To evaluate the real-time feasibility of the proposed framework, the end-to-end runtime of the EKF-based full estimation pipeline was analyzed. All experiments were conducted on a system equipped with an AMD Ryzen 5 5600X 6-core Processor (Advanced Micro Devices, Santa Clara, CA, USA) and an NVIDIA GeForce RTX 3060 Ti GPU (NVIDIA Corporation, Santa Clara, CA, USA). Table 5 summarizes the mean and standard deviation of the runtime across 5 different sequences. For each sequence, the average runtime was computed by dividing the total wall-clock execution time of the main filtering loop by the total number of processed IMU steps. The standard deviation was calculated to quantify the variability of runtime across steps. For all evaluated sequences, the mean runtime per step was measured to below 3 ms, demonstrating stable and consistent computation performance. These results indicate that proposed EKF-based radar inertial odometry satisfies the real-time requirements of autonomous robotics systems.

Although this work adopts a scan-to-scan ICP formulation, extending the framework to longer temporal windows, such as scan-to-submap registration, is one of the planned directions for future work. This is because such an extension can not only improve overall navigation accuracy but also help mitigate the degradation of ICP registration quality in challenging scenarios, for example when the radar frame rate is low or when the sensor platform undergoes aggressive maneuvers. When a submap composed of scans collected at multiple time instances is employed, additional design considerations are required for state augmentation and reference-state definition. Therefore, when multiple temporal observations are aggregated within a submap representation, a principled formulation is required to define and select an appropriate reference state. A systematic treatment of submap-based state representation and reference-state definition will be addressed in future work.

## 5. Conclusions

This paper presents an ICP-based RIO framework for 4D imaging radar and further extends it by integrating Doppler velocity measurements to construct the INS/ICP/Ego-velocity architecture that fully utilizes position, velocity, and attitude information. In other words, by proposing a consistent filter-based fusion framework that integrates both radar point cloud registration (ICP) and Doppler velocity, this study established an RIO system that effectively integrates all available radar observations.

For performance evaluation, three configurations—using only ICP, only Doppler velocity, and the proposed algorithm that uses both ICP and ego-velocity as measurements—were compared across various sequences from two open datasets. All evaluations were conducted using the Evo library [32] under identical experimental conditions. The results demonstrated that the proposed algorithm that uses both ICP and ego-velocity as measurements, which exploits all available measurements, achieved superior accuracy and robustness in most sequences. This confirms that integrating complementary information from point cloud registration and Doppler velocity leads to improved estimation stability and consistency compared to single-source approaches. The proposed ICP-integrated observation matrix was designed to accommodate various state-of-the-art ICP algorithms, ensuring scalability and flexibility of the framework. Owing to its modular design, the ICP component can be readily replaced or upgraded within the same filter structure, enabling seamless integration of future registration techniques without modifying the overall system architecture.

Future work will also focus on enhancing the robustness of the overall pipeline. A preprocessing module will be developed to perform dynamic object removal using Doppler velocity and to incorporate additional state variables for online calibration within the filter. These enhancements are expected to further improve noise reduction performance, maintain measurement consistency, and strengthen filter convergence stability throughout the processing pipeline. Such extensions will preserve the advantages of the proposed framework while enhancing its applicability and reliability for real-world, real-time navigation scenarios.

## Figures and Tables

**Table 1:** Detailed ICP parameter Configuration. ρ: Kernel Value, γ: Constraint Weight, N: Maximum Iteration Number, τ: Convergence Threshold.

ρ	γ	N	τ
0.6	0.2	60	1 × 10^−6^

**Table 2:** Summary of the Selected Sequences from Dataset.

	Type	Weather /Lighting	Distance [km]	During [s]
20231208/1	Urban	Clear/Day	0.3	147
20231213/1	Urban	Light rain/Night	0.3	126
Library03	Urban	Snow/Day	0.8	156
Parking Lot04	Urban	Snow/Day	0.4	112
Sports Complex02	Urban	Cloud/Night	0.7	143

**Table 3:** Quantitative result on Snail Dataset.

	Method	RTE [m]	RRE [°]	σ [m]	σ [°]	ATE [m]
		Mean/RMSE	Mean/RMSE	Mean/RMSE	Mean/RMSE	Mean/RMSE
20231208/1	ICP	0.031/0.043	0.037/0.068	0.030	0.056	1.272/1.700
	ICP/EV	0.027/0.038	0.043/0.077	0.027	0.064	2.215/2.743
20231213/1	ICP	0.036/0.041	0.032/0.049	0.038	0.019	1.686/2.010
	ICP/EV	0.035/0.040	0.029/0.045	0.019	0.034	1.643/2.000

**Table 4:** Quantitative result on HeRCULES Dataset.

	Method	RTE [m]	RRE [°]	σ [m]	σ [°]	ATE [m]
		Mean/RMSE	Mean/RMSE			Mean/RMSE
Library03	EKF-LC	0.063/0.094	0.033/0.061	0.070	0.050	3.358/3.685
	Kiss-ICP	0.061/0.103	0.034/0.063	0.069	0.054	5.112/5.345
	4DRadarSLAM	0.108 /0.138	0.092/0.099	0.122	0.099	18.76/30.01
	Proposed	0.061/0.087	0.034/0.059	0.062	0.048	2.872/3.295
Parking Lot04	EKF-LC	0.108/0.142	0.041/0.060	0.092	0.043	2.622/2.881
	Kiss-ICP	0.155/0.183	0.103/0.124	0.091	0.069	3.168/3.268
	4DRadarSLAM	1.172/1.629	1.189/1.766	1.113	2.020	36.77/44.74
	Proposed	0.110/0.138	0.041/0.059	0.091	0.042	2.780/3.051
Sports Complex02	EKF-LC	0.253/0.332	0.220/0.254	0.188	0.319	18.82/22.92
	Kiss-ICP	0.110/0.140	0.043/0.062	0.090	0.100	3.361/3.753
	4DRadarSLAM	0.198/0.288	0.198/0.213	0.187	0.145	11.82/13.20
	Proposed	0.098/0.114	0.054 /0.079	0.058	0.059	4.950/5.287

**Table 5:** Runtime Statistics of the Proposed Pipeline.

	Mean Runtime [ms]	Std [ms]
20231208/1	2.110	0.729
20231213/1	2.165	0.862
Library03	2.245	1.899
Parking Lot04	1.368	0.984
Sports Complex02	1.237	0.859

## Data Availability

Restrictions apply to the availability of these data. Data were obtained from the Snail radar dataset and HeRCULES dataset and are available from Huai et al. [30] at https://github.com/snail-radar/snail-radar.github.io (accessed on 3 September 2025) and Kim et al. [31] at https://sites.google.com/view/herculesdataset (accessed on 6 October 2025), and are available with the permission of the original Huai et al. [30] and Kim et al. [31].
